# Redistributive effects of the National Health Insurance on physicians in Taiwan: a natural experiment time series study

**DOI:** 10.1186/1475-9276-12-13

**Published:** 2013-02-04

**Authors:** Chiang-Hsing Yang, Yu-Tung A Huang, Ya-Seng A Hsueh

**Affiliations:** 1 Department of Health Care Management, National Taipei University of Nursing and Health Sciences, Taipei, Taiwan; 2 Department of Gerontological Care and Management, Chang Gung University of Science and Technology, Tao-Yuan, Taiwan; 3 Centre for Health Policy, Programs and Economics, Melbourne School of Population and Global Health, The University of Melbourne, Level 4, 207 Bouverie Street, Carlton, 3053 Victoria, Australia

## Abstract

**Background:**

Previous studies have evaluated the effects of various health manpower policies but did not include full consideration of the effect of universal health insurance on physician re-distribution. This study examines the effects of implementing National Health Insurance (NHI) on the problem of geographic mal-distribution of health providers in Taiwan.

**Methods:**

Data on health providers and population between 1971 and 2001 are obtained from relevant governmental publications in Taiwan. Gini coefficients derived from the Lorenz curve are used under a spline regression model to examine the impact of the NHI on the geographic distribution of health providers.

**Results:**

The geographic distribution equality of the three key health providers has improved significantly after the implementation of NHI program. After accounting for the influences of other confounding factors, Gini coefficients of the three key providers have a net reduction of 1.248% for dentists, 0.365% for western medicine physicians, and 0.311% for Chinese medicine physicians. Overall, the absolute values of the three key providers’ Gini coefficients also become close to one another.

**Conclusions:**

This study found that NHI’s offering universal health coverage to all citizens and with proper financial incentives have resulted in more equal geographic distributions among the key health care providers in Taiwan.

## Background

During the past three decades Taiwan has experienced an economic miracle that has led to a substantial increase of resources in virtually every aspect of the society, including the number of physicians relative to the population continues to grow. However, as many countries worldwide, the distribution of physician manpower in Taiwan has remained unequal with some areas getting far more physicians than others [1-7]. It has been a priority for the Taiwan government to combat this mal-distribution problem.

The Taiwan government has adopted many strategies such as a healthcare network plan since 1985. It was purported to reach at least one physician per 3,000 people in rural areas by increasing medical schools to train more physicians. As a result, the number of active physicians had steadily increased from 5.4 per 10,000 citizens in 1957 to 10.3 per 10,000 in 1991, and 16.77 per 10,000 in 2010 [8]. However, Kobayashi and Takaki [1] have found that, in Japan increasing medical students alone cannot improve the equal geographic distribution of physicians. This is also the case in Taiwan. More physicians had not resulted in better access for the patient, because physicians are remained concentrating in metropolitan and urban areas during these three decades.

The implementation of National Health Insurance (NHI) program in 1995 has been of paramount importance in this regard. The NHI has provided a unique opportunity for us to observe whether this intervention has impacted on the distribution of health care manpower over geographic areas. Taiwan’s NHI is characterized by mandatory enrollment for all citizens, a government-run insurer, a single-payer system, and comprehensive benefits coverage (including dental care and Chinese medicine). From the beginning, NHI has extended the enrollment rate from 54% in 1994 to over 96% of the entire population in Taiwan in 1995, and has maintained this high coverage level since then.

The contract rates of three key health providers, namely western medicine physicians, Chinese medicine physicians and dentists with the Bureau of NHI have also remained high since the implementation of NHI. As of December 2000, the contract rates had already reached as high as 88.04%, 84.99%, and 96.13%, respectively for western medical clinics, Chinese medical clinics, and dental clinics. In addition, the implementation of NHI has achieved universal coverage by providing greater medical care access for citizens in Taiwan [9].

Given equity of health is important, previous studies have explored the financial fairness to health care [10-12], the equity access to health care [13-15], the equity distribution of medical resources [4,5,16-18], and the effects of various health manpower policies on improving physician mal-distribution [1,3,5-7,19-21]. Few studies have examined whether universal health insurance provisions is associated with more even geographical distribution of physicians. Therefore, this study aims to investigate the effects of implementing National Health Insurance (NHI) on the geographic mal-distribution of western medicine physicians, Chinese medicine physicians and dentists in Taiwan with two objectives. One objective is to examine the changes in geographic distribution of physicians before and after the implementation of NHI, by analyzing the trends of distribution of western medicine physicians, Chinese medicine physicians and dentists over the past three decades. The other objective is to examine whether NHI has affected the geographic distributions of these three types of health providers differently.

## Methods

This study is based on a natural experiment. The intervention is the implementation of NHI in Taiwan from March 1, 1995. There are three experimental groups, namely western medicine physicians, Chinese medicine physicians and dentists. Since NHI covers all three types of physicians, there is no control group available for this study. In order to reduce the potential bias due to self-selection of the experimental groups, this study has employed an interrupted trend analysis with time series observations for 32 years, including 24 year before NHI and 8 years after NHI (including 1995). The three experimental groups are thus able to serve as their own control by the long term “before” versus “after” trend analysis. The reason of choosing the years of observation is explained later in this section.

The equality of geographic distributions for each of the three types of health providers is measured by Gini coefficients derived from Lorenz curve based on 21 prefecture/city assigned by the Ministry of Interior. The Lorenz curve and Gini coefficients are developed primarily for measuring the degree of unequal distribution of income within a population [13,22,23]. A Lorenz curve of cumulative wealth or income versus population, for instance, plots cumulative population in ascending order on the X-axis against the cumulative wealth or income in those areas on the Y-axis. The Gini coefficient is derived from the Lorenz curve that provides an alternative numerical measure for the extent of equal distribution in wealth or income. Its value lies between 0 (no inequality) and 1 (complete inequality). These methods have been applied for studying the equal distribution of physicians [1,2,7,24-26].

The dependent variable Gini coefficient in this study is a summarized result of the accumulation the number of physicians against the accumulation of population of all the 21 geographic areas (in the order of physician concentration in a prefecture/city) compared to perfect distribution of physicians (i.e., for a certain percentage of population it has the same percentage of physicians. For example, 40% of population has 40% of physicians, 70% of population has 70% of physicians, accumulated from the lowest to the highest ratio of physicians to population in an area).

The spline-regression analysis is developed for determining whether there is a structural change in the Gini coefficients due to external program intervention over time, such as policy changes. This is different from regression with dummy variables, which assumes one time change in a linear form. Spline regression employs the polynomial term for capturing non-linear changes along with the introducing of the policy [27]. This specific econometric model has been introduced to the health care research in recent years [28]. Since NHI was implemented to cover the entire health care system in Taiwan, all the relevant parties within the health care system needed a substantial period of time to adjust into the NHI scheme. In particular, a period of time had been required for the relocation or redistribution of physicians, clinics or hospitals. It was impossible to result in an immediate stable 'before versus after' effect due to the implementation of NHI. It is more likely that such effect of NHI is gradual. Therefore, in our study we have employed a spline-regression, using 0 for the variable *NHI*_*t*_ before 1995, and 1 to 8 after 1995 to capture potential non-linear structural change after the implementation of NHI.

Given three decades is a long period of time, with other confounding socio-economic factors in the wider society, the total difference between the 'pre' and 'post' NHI period cannot be attributed totally to the effect of NHI. Therefore, a year specific time trend variable denoted by the term T_*t*_, is included in our model for capturing and controlling for the natural growth effect each year due to all other confounding factors between the pre and post NHI periods.

After the inception of NHI, several major health insurance measures on physician payment have been adopted by the Government after 2001, for example pay-for-performance in 2001; global budgeting in 2002; and regressive payment to service volume for assuring quality of care in 2004. All these policies and measures have been co-existing since each of them was added into the system. Including these measures would have brought in major confounding effects. This was why we have only included our observations to the years up to 2002 but still allowed for a solid 8-year observation for the trend after NHI.

The spline-regression model of this study can be shown in the equation below: *G*_*t*_ = *I* + *β*_1_*T*_*t*_ + *β*_2_*NHI*_*t*_ + *ε*_*t*_

where

*G*_*t*_: the Gini coefficient of *t*^th^ year

*I*: Intercept

*T*_*t*_: time trend (between 24 periods before NHI to 8 periods after NHI)

***NHI***_***t***_**(spline point)**: 0, the period before NHI

otherwise, the year^th^ after implemented NHI (between 1 to 8)

In this study, data on geographic locations of health professionals were drawn from the Health and Vital Statistics, which is published annually by the Department of Health. Data on population were obtained from the Taiwan-Fukien Demographic Fact Book, which is published by the Ministry of the Interior. These data covered the period between 1971 and 2002. Currently, Taiwan is divided into 21 prefecture/cities. The accumulative percentage of health providers to population for each medical resource area and each year was calculated in order to obtain the Gini coefficient of geographic distribution for each of the three types of health providers in every year from 1971 to 2002.

In addition to the Gini coefficients, western medicine physicians to population ratios over time were also calculated for comparison to address the issue concerning the NHI’s impact on the geographic distribution of western medicine physicians. Given Taiwan utilizes a closed health care staff system, western medicine physicians are either employed full time by hospitals (i.e., hospital-based physicians) or practice independently in their own clinics (i.e., clinic-based physician). Trends representing the number of hospital-based and clinic-based western medicine physicians were also used to examine the potential impact of NHI on physician distribution between these two settings. Due to the constraints of data, both western medicine physician to population ratio and the number of hospital-based or clinic-based western medicine physicians are available only from 1986 and thereafter.

## Results

Table 1 presents the changes in distribution (measured in Gini Coefficients) of three types of physicians in Taiwan for selected years over the period between 1971 and 2002 and their ratios to population. The ratio of physicians to population had increased stably from 1970s to 1980s, increased drastically from 1981 to 1991, and increased slowly but stably toward 2002. The ratio of Chinese medicine physicians to population had decreased slightly between 1971 and 1981, increased slowly through the beginning of the inception of NHI and increased fast from then on. The ratio of dentists to population had almost doubled between 1971 and 1981, and increased even faster from 1981 to 1991. This ratio had increased substantially between 1991 and 2002.

**Table 1:** Physician to population ratios and Gini coefficients of three types of physicians in Taiwan for selected years

**Year**	**1971**	**1981**	**1991**	**1996**	**2001**
Population	14,937,054	18,193,955	20,605,831	21,525,433	22,405,568
Western medicine physicians					
Number	6,375	11,957	21,115	24,790	30,562
per 100,000	42.68	65.72	102.47	115.17	136.40
Gini coefficient	0.25	0.25	0.26	0.26	0.25
Chinese medicine physicians					
Number	1,466	1,682	2,514	2,992	3,979
per 100,000	9.81	9.24	12.20	13.90	17.76
Gini coefficient	0.26	0.26	0.22	0.20	0.19
Dentists					
Number	910	2,128	5,983	7,254	8,944
per 100,000	6.09	11.70	29.04	33.70	39.92
Gini coefficient	0.32	0.39	0.35	0.31	0.26

**Figure 1:**
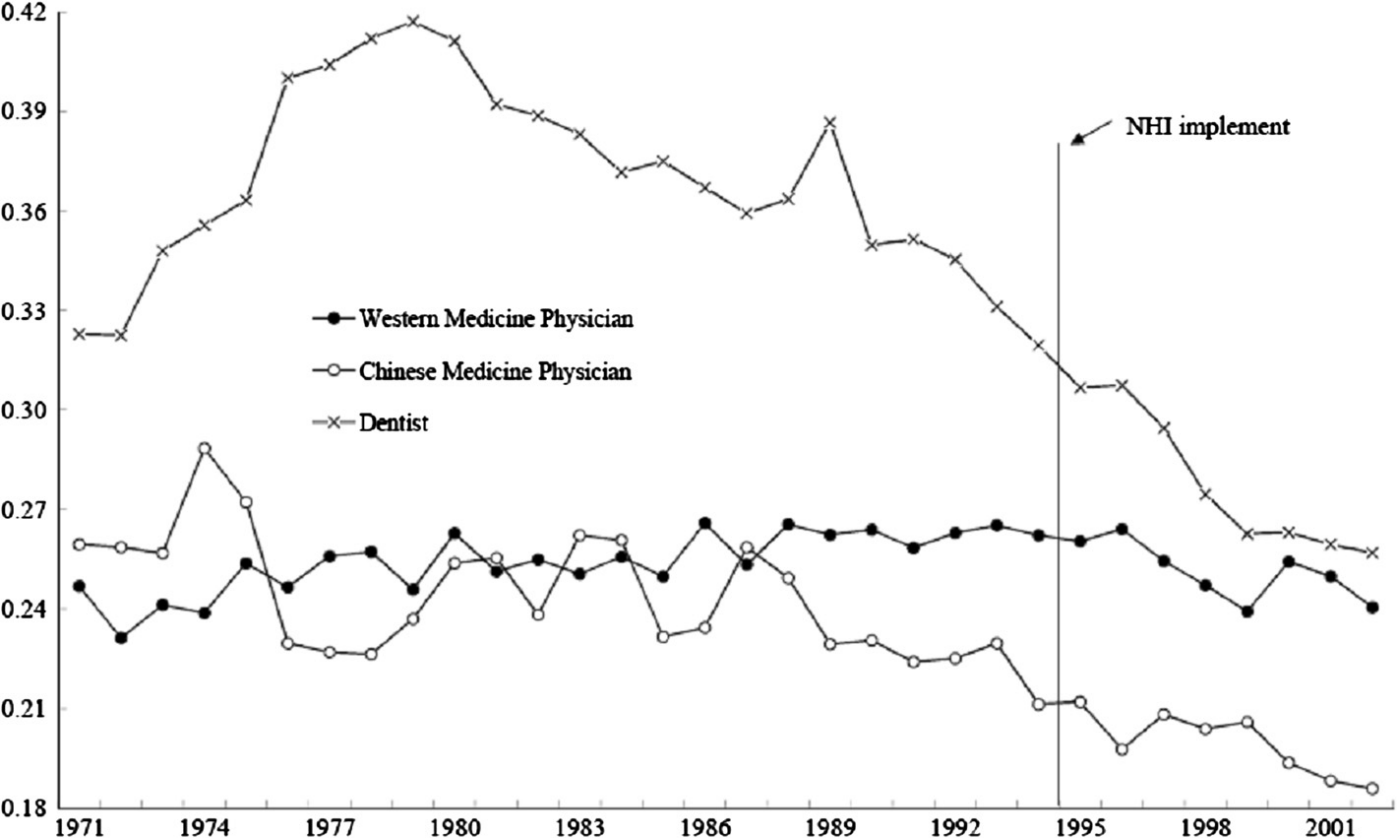
The trends of Gini coefficients of geographic distribution for each of the three types of physicians in Taiwan, 1971–2002.

**Figure 2:**
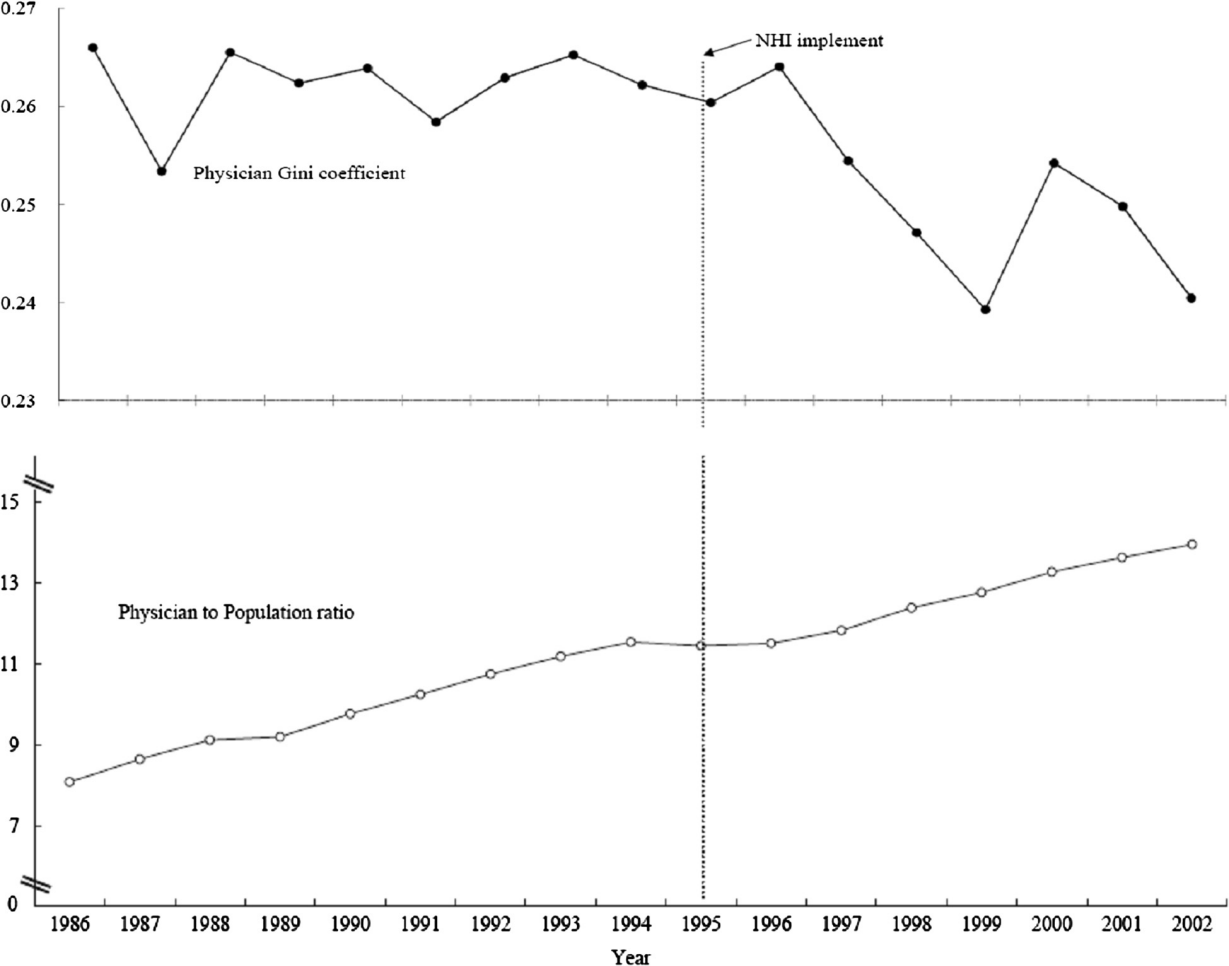
Western medicine physician to population ratio vs. Gini coefficients of geographic distribution of western medicine physician in Taiwan, 1986–2002.

All yearly Gini coefficients from 1971 to 2002 were plotted and presented in Figure 1. The gray line in the middle of the figure represents the Gini coefficient of western medicine physicians each year for the period of 32 years. Figure 1 shows that, even with changes in health manpower policies, the inequality of geographical distribution of western medicine physicians had basically remained unchanged or even worsened before NHI. Figure 2 shows that the ratio of active western medicine physicians to population had continued to increase throughout the observation period, for both before and after the installation of NHI. Thus, increasing the absolute number of physicians had not resulted in a more even geographic distribution of the western medicine physicians. On the other hand, Figure 1 indicates that both of the Gini coefficients of dentists and Chinese medicine physicians show a somewhat declining trend since 1990, and appear to have declined more rapidly after NHI.

The above graphs’ results reveal that the inequality of geographic distribution had declined significantly after the implementation of NHI – regardless of the trend patterns of geographic distribution, for all three types of health care providers before NHI (see Figure 1). Furthermore, statistical tests presented in Table 2 show that, after controlling for the natural growth by the time trend, the changes of Gini coefficients for western medicine physicians, dentists, and Chinese medicine physicians are all negative and statistically significant. This provides concrete support to the findings of this study. That is, the mal-distributions of all three types of health care providers have a negative trend. The improvements in equality of geographic distribution in terms of Gini coefficient are by 1.3% for dentist, 0.4% for western medicine physician, and 0.3% for Chinese medicine physician. Furthermore, the absolute values of the three key health providers’ Gini coefficients also became closer to one another (see Table 1).

**Table 2:** Spline regression results of impacts of NHI on the Gini coefficients for the geographic distribution of three types of physicians in Taiwan, 1971-2002

	**Western medicine physicians**			**Chinese medicine physicians**			**Dentists**		
	**β (s.e)**			**β (s.e)**			**β (s.e)**		
Intercept	0.265 (0.002)^†^			0.223 (0.005)^†^			0.350 (0.010)^†^		
Time trend	0.001 (0.000)^†^			−0.002 (0.000)^†^			−0.001 (0.000)		
NHI	−0.004 (0.001)^†^			−0.003 (0.002)^*^			−0.013 (0.003)^†^		
F-value	20.854^†^			38.295^†^			33.245^†^		
*R*_*adj*_^2^	0.562			0.706			0.675		

* *p*<0.05; ^†^*p*<0.01.

**Figure 3:**
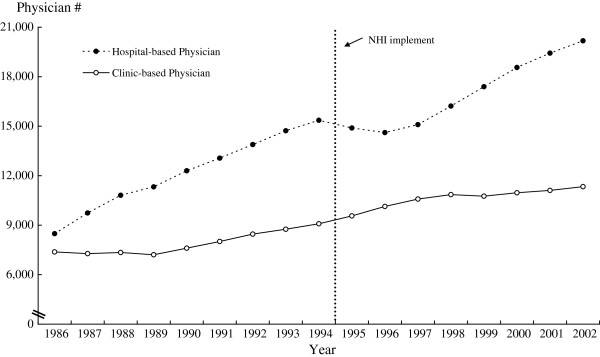
Trends of hospital-based vs. clinic-based western physicians in Taiwan, 1986–2002.

The trend of the number of western medicine physicians employed full time by hospitals versus practicing independently in their own clinics (see Figure 3) provides additional information to support the possibility for a more equal geographic distribution of western medicine physicians due to the implementation of NHI. From the beginning of the observation period in 1986, the number of (independent) clinic-based physicians had increased slightly and steadily, yet had tended to increase faster after the implementation of NHI. On the other hand, the number of hospital-based physicians had increased much faster from 1986 until the time before the implementation of NHI. The trend had encountered a downward pattern for about three years before increasing again. It appears that more western medicine physicians went to practice independently in clinics rather than served in hospitals immediately after the implementation of NHI. The phenomenon of physicians practiced in hospitals increased again after three years of NHI will be discussed in the next section.

## Discussion

There are three major findings of this study that are worthy to be discussed. Firstly, the implementation of national health insurance in Taiwan has been associated with a noticeable improvement of the distribution of all three types of key health care providers. In Figure 1, the Gini coefficients of western medicine physician show relative little improvement before the implementation of NHI, but declined after the introduction of NHI. The significance of this change is further confirmed by the statistical tests shown in Table 2. The findings in this study are consistent with those from other studies [1,25].

Two factors are most likely to explain why NHI in Taiwan had created a more even geographic distribution of western medicine physicians. One factor is the universal coverage nature of NHI. It has increased and equalized medical care affordability or purchasing power of the patients in Taiwan. Before the advent of NHI, people with adequate health care purchasing power were more likely living in the metropolitan or urban areas. This in turn might have attracted more health care providers to locate in those areas. After the installation of national health insurance with a coverage rate of 96%, NHI provides almost all patients with similar ability to pay for health care services around the country. As a result, effective demand is no longer strongly concentrated in the metropolitan or urban areas. This change may have attracted health care providers to move into previously underserved areas. This phenomenon suggests that, in order to enhance a more even supply of physicians, policy makers may want to increase as well as equalize the purchasing power of the consumers so that the demand-pull effect can enhance or redirect the supply of key health care providers to the needy areas throughout the country.

The other factor is the financial incentives that NHI has provided to physicians, especially for the western medicine physicians. For example, in 1996, NHI fees for physician visits were made 19-33% higher than the Labor Insurance fee schedule under the pre NHI period [9,29]. Physician fees (all three types of physicians) for providing ambulatory and emergency services were also higher than those of providing inpatient services. For example, in 1996, the reimbursement rate for ambulatory visit increased from NT $245 to $333 per visit after the implementation of NHI, representing 17–34 % higher than the inpatient physician fees. This might have created a strong financial incentive for all three types of physicians to practice in their own clinics rather than in a hospital. This is because under the close-hospital system in Taiwan the hospital-based physicians, who are the employees of the hospital, have to share the reimbursed revenue with the hospital; whereas the clinic-based physicians, who are the owner of the clinic, can receive full incremental reimbursement from NHI’s change in fee scheme. As a result, providers who practice in hospitals have not obtained the same rate increase as the clinic-based providers.

In addition, under the tax codes [30], 72% of NHI’s reimbursement is tax exempt and the remaining 28% is taxable income, regardless of the location where a physician practices. This tax treatment tends to favour all three types of physicians practicing in non-urban areas where the living and practice costs are generally much lower than those costs in urban areas. Thus, the tax incentive is likely to induce health care providers practice in rural area.

The second major finding is that the extent of equality distribution improvement although varied in magnitudes, the trend is toward closer to one another for the western medicine physicians, dentists, and Chinese medicine physicians as a result of the implementation of the NHI program. The improvement of the distribution of dentists was the highest among the three types of health providers in Taiwan. This was because the mal-distribution of dentists was the most serious among the three before NHI. The Gini coefficients of dentists (see Figure 1) although are still the highest among the three types of physicians, it became close to that of the western medicine physicians after the implementation of NHI. The geographic distribution of Chinese medicine physicians has also improved more than that of the western medicine physicians. This phenomenon could possibly be explained by the theory of resources dependence. This theory maintains that an individual or an organization will seek to develop linkage with other organizations possessing resources that are critical to their survival and development [31]. Dentists and Chinese medicine physicians usually care for less urgent and less fatal patients. They are less dependent on advanced medical equipments and infrequently refer their patients to hospitals. In contrast, western medicine physicians in clinics are able to care for patients with complex or urgent medical conditions by referring them to hospitals Therefore, western medicine physicians are expected to be more dependent on hospitals for their practice, compared to dentists and Chinese medicine physicians. Thus, more western medicine physicians tend to practice in urban areas within hospitals or in their clinics nearby hospitals than their dentists and Chinese medicine physicians counterparts. Another possible explanation is the “spill-over effect”. Toyokawa and Kobayashi found that the increasing supply of dentists in Japan supports the “spill-over” hypothesis, which predicts geographic diffusion of dentists toward less competitive area. In contrast, increasing the number of physicians had resulted in more concentrated geographic distribution of physicians in urban areas [32].

The third finding is that we found there was a fluctuation in the improvement of distribution of western medicine physicians after NHI. Figure 3 shows that, after 1997 the growth rate of hospital-based physicians was much faster than that of clinic based physicians. One way of interpreting this pattern may be that the mal-distribution of physicians had even more deteriorated after 1997. However, the statistical test results in Table 2 indicate an improvement of physician distribution after NHI, even though the growth of hospital-based physicians seemed faster than that of clinic-based physicians. This means that, the western medicine physicians distributed more evenly while more physicians were working in the hospital. This seemingly contradictory phenomenon suggests there could be an increase in the number of hospitals or hospital beds in under served areas in Taiwan.

In fact, our yearly prefecture-specific data (too much to be presented in this article) indicate that the number of hospitals relocated to under served areas was indeed gradually increasing after NHI, particularly after 1997 to cash in the NHI’s guaranteed reimbursement to the hospitals for outpatient consultations. In other words, hospital-based physicians were increasing after NHI, and more of those physicians had worked at hospitals in under served remote areas after NHI. A more even allocation of hospitals has contributed to a more even distribution of physicians. Therefore, directing more hospitals to open in medically under-served areas and/or increasing beds of existing hospitals in those areas can increase the equalization of the distribution of physicians by attracting more physicians to practice in under-served areas.

This study has several limitations. First, the effects of possible cross substitutions between western medicine and Chinese medicine physicians are not evaluated in this study. Cross substitutions might have affected the distribution of western medicine physicians. However, even if the effect of the substitution factor did occur, it is likely to only strengthen the results of this study. This is because, according to the findings of this study, the Chinese medicine physicians have been more evenly distributed after NHI than the western medicine physicians and dentists. This may give a stronger competition pressure towards western medicine physicians and cause a negative impact on the distribution equality of western medicine physicians. Even so, we still have observed a significant decrease of the distribution inequality of western medicine physician after NHI.

Secondly, analyzing the overall western medicine physician population distribution is only one measurement. This study did not indicate which specialists are more likely to be influenced by an insurance reimbursement scheme or health policy. In fact, after implementing the NHI program, there is also a significant distribution imbalance of medical specialists such as OB/GYN and others. Therefore, when relevant data are available, further research could examine the relationship between geographic distribution of specialized physicians and NHI.

## Conclusions

According to Taiwan’s experience, before the installation of NHI, manpower policies of increasing the quantity of providers had not resulted in a more equalized physician geographic distribution. In contrast, NHI’s offering universal health coverage to all citizens and proper financial incentives to providers have resulted in more equal geographic distributions among western medicine physicians, Chinese medicine physicians and dentists in Taiwan. Moreover, the extent of equality distribution improvement although varied in magnitudes, the trend is toward closer to one another for the western medicine physicians, dentists, and Chinese medicine physicians as a result of the implementation of the NHI program.

## Competing interests

The author(s) declare that we have no competing interests.

## Authors’ contributions

CHY conceived of the study, and participated in its design, coordination, and draft the manuscript. YtAH participated in the design of the study and performed the statistical analysis. YsAH carried out the studies, participated in the study design, guided the statistical analysis, and drafted the manuscript. All authors read and approved the final manuscript.
